# Adipocytes and macrophages secretomes coregulate catecholamine-synthesizing enzymes

**DOI:** 10.7150/ijms.52219

**Published:** 2021-01-01

**Authors:** Andreia Gomes, Fernanda Leite, Laura Ribeiro

**Affiliations:** 1 Department of Biomedicine, Unit of Biochemistry, Faculty of Medicine, University of Porto. Alameda Prof Hernâni Monteiro, 4200-319 Porto, Portugal.; 2 i3S, Instituto de Investigação e Inovação em Saúde, University of Porto. Rua Alfredo Allen 208, 4200-135 Porto, Portugal.; 3 Department of Clinical Haematology, Centro Hospitalar Universitário of Porto, Largo Professor Abel Salazar, 4099-001, Porto, Portugal.; 4 UMIB/ICBAS - Unit for Multidisciplinary Investigation in Biomedicine- Institutode Ciências Biomédicas Abel Salazar, University of Porto, Porto, Portugal.; 5 Department of Public Health and Forensic Sciences, and Medical Education, Faculty of Medicine, University of Porto. Alameda Prof Hernâni Monteiro, 4200-319 Porto, Portugal.

**Keywords:** catecholamines, adipose tissue, adipocytes, macrophages, secretome, inflammation

## Abstract

Obesity associates with macrophage accumulation in adipose tissue where these infiltrating cells interact with adipocytes and contribute to the systemic chronic metabolic inflammation present in immunometabolic diseases. Tyrosine hydroxylase (TH) and phenylethanolamine N-methyltransferase (PNMT) are two of the main enzymes of catecholamines (CA) synthesis. Adipocytes and macrophages produce, secrete and respond to CA, but the regulation of their synthesis in the interplay between immune and metabolic systems remains unknown. A model of indirect cell coculture with conditioned medium (CM) from RAW 264.7 macrophages with or without LPS-activation and 3T3-L1 adipocytes and preadipocytes was established to study the effect of cellular secretomes on the expression of the above enzymes. During the adipocyte differentiation process, we found a decrease of TH and PNMT expression. The secretome from LPS-activated macrophages downregulated TH and PNMT expression in preadipocytes, but not in mature adipocytes. Mature adipocytes CM induced a decrease of PNMT levels in RAW 264.7 macrophages. Pre and mature adipocytes showed a similar pattern of TH, PNMT and peroxisome proliferator-activated receptor gamma expression after exposure to pro and anti-inflammatory cytokines. We evidenced macrophages and adipocytes coregulate the expression of CA synthesis enzymes through secretome, with non-inflammatory signaling networks possibly being involved. Mediators released by macrophages seem to equally affect CA production by adipocytes, while adipocytes secretome preferentially affect AD production by macrophages. CA synthesis seems to be more determinant in early stages of adipogenic differentiation. Our results suggest that CA are key signaling molecules in the regulation of immune-metabolic crosstalk within the adipose tissue.

## Introduction

Obesity and related immunometabolic diseases are systemic low-grade inflammatory conditions 1, 2. The excessive increase of body fat mass is a major trigger for adipose tissue (AT) remodeling, leading to the recruitment and activation of immune cells 3. In obesity, both adipocytes and macrophages are prone to develop inflammation by increasing the secretion of pro-inflammatory cytokines 4, 5. Previous studies reported that cellular secretome signature as well as signaling immune receptors are altered in obesity 6, 7 and contribute to the pathophysiology of metabolic diseases 8.

Immune cells, neurons and adipocytes seem to share signaling pathways mediated by catecholamines (CA) through the activation of adrenoceptors (AR) 9, 10 and dopaminergic receptors present in these cells 11-13. Moreover, endogenous CA through autocrine/paracrine mechanisms modulate inflammatory responses in monocytes and other immune cell types 10, 14.

The synthesis of CA relies on the sequential enzymatic processes upon the amino-acid tyrosine herein with special emphasis on tyrosine hydroxylase (TH), the rate-limiting step, and phenylethanolamine N-methyltransferase (PNMT) that converts NA to AD 15.

The interplay between adipokines and CA 9 suggests a role for these molecules linking obesity to inflammation. Indeed, in rat visceral AT lipopolysaccharides (LPS) and immobilization insults modulate the immune response and increase macrophage infiltration associated with endogenous CA production 10. CA signaling in macrophages has been reported to modulate inflammation by decreasing TNF-α secretion 14.

We hypothesize that, under an obesogenic state, CA play a role in the crosstalk between metabolic and immune systems. In this study, we investigated the effect of secreted factors from LPS‐activated macrophages and murine adipocytes on the regulation of TH and PNMT expressed in both macrophages and adipose cells. The present work aims to deepen the knowledge about the pathophysiological role of endogenous CA as newly described signaling pathways in immunometabolism.

## Methodology

### Cell line cultures

Mouse 3T3-L1 preadipocytes and RAW 264.7 murine macrophage cell lines were obtained from the ATCC® CL-173™ and TIB™, respectively. Preadipocytes were maintained in Dulbecco's modified Eagle's medium (DMEM; Sigma-Aldrich, Germany) with 4.5 g/L glucose, supplemented with 4mM L-glutamine, 1% (v/v) antibiotic-antifungal (Penicillin, Streptomycin and Amphotericin B, Sigma-Aldrich, Germany), 3.7 g/L of sodium bicarbonate (Sigma-Aldrich, Germany) and 10% (v/v) heat-inactivated bovine neonate serum (NCS) Sigma-Aldrich, Germany). RAW 264.7 cells were cultured in DMEM supplemented with 10% (v/v) of heat-inactivated fetal bovine serum (FBS, Sigma-Aldrich, Germany) and 1% antibiotic-antifungal (v/v). Cells were cultured and maintained in a humidified atmosphere of 5% CO2 at 37°C in a standard incubator. Cultures were passaged every 2-3 days, when 80% confluence was reached. The obtained cells were exposed to a cell viability test with trypan blue and subsequently cultured in the appropriate support according to the purpose of the experiment. All treatments were conducted in serum-free conditions.

#### 3T3-L1 differentiation and conditioned medium collection

3T3-L1 preadipocytes were seeded at a density of 4 000 cells/cm^2^ and kept in standard growth medium. Four days after confluence, differentiation was induced with differentiation medium I (DMEM with 4.5 g/L of glucose supplemented with L-glutamine (4 mM), an antibiotic-antifungal solution 1% (v/v), bicarbonate (1.5 g/L), FBS 10% (v/v) (PAA, Pasching, Austria), 10 μg/mL insulin, 0.25 μM dexamethasone and 0.5 mM isobutylmethylxanthine (IBMX) (Sigma- Aldrich, Germany). After three days, this medium was replaced by differentiation medium II (DMEM with glucose (4.5 g/L) supplemented with L-glutamine (4 mM), 1% (v/v) antibiotic-antifungal solution, sodium bicarbonate (3.7 g/L), bovine fetal serum (10% v/v) and insulin (10 μg/mL), which was thereafter renewed every 2 days. Experiments were performed at day 0, 3, 6 and 10 after the induction. After days 0 and 10 of differentiation, cells were grown in serum-free medium for 24 h and conditioned medium was later collected, centrifuged at 10 000 g for 5 min and the supernatant stored at -20°C for subsequent experiments.

#### Raw 264.7 activation and conditioned medium collection

Cells were incubated for 24 h with lipopolysaccharide (LPS) from Escherichia coli K12 (InvivoGen, San Diego, CA) at 500 ng/mL to induce macrophage activation as previously described 16, 17. Subsequently, LPS-activated RAW 264.7 macrophages and RAW 264.7 macrophages were grown in serum-free medium that was later collected, centrifuged at 10 000 g for 5 min and the supernatant stored at -20°C for subsequent experiments.

### Oil Red O staining and quantification

Oil Red O (Sigma-Aldrich, Germany) was used to stain intracellular lipid droplets in differentiated adipocytes. Oil Red O was solubilized in 100 μL 100% (v/v) isopropanol and the absorbance measured at 510 nm using a microplate reader (Thermo Scientific, Multiskan Ascent Plate Reader).

### Evaluation of gene expression by qRT-PCR

The relative expression (mRNA levels) of TH and PMNT involved in CA synthesis was determined by qRT-PCR. The TriPure isolation reagent (Roche Diagnostics, Germany) was used to extract total RNA from cells, according to the manufacturer's protocol. For cDNA synthesis, 2 µg of RNA were incubated at 37°C for 30 minutes with DNase I, RNase-free (1U/µL) RiboLock RNase inhibitor (20 U/µL), 5X First-Strand Buffer (250 mM Tris-HCl, pH 8.3, 20 mM MgCl_2_, 50 mM DTT). DNase was denatured 15 minutes at 65°C, and 0.2 µg/µL of random hexamers was added to the mix and incubated 5 minutes at 65°C. Later, 10mM solution of deoxyribonucleotide triphosphate, RiboLock RNase inhibitor (20 U/µL) and Reverse Transcriptase (200 U/µL) were added and incubated 5 minutes at 25°C, 60 minutes at 42°C and denatured 5 minutes at 70°C, placed on ice and stored. All reagents were obtained from Thermo Scientific (Waltham, USA). After cDNA synthesis, TH, PNMT and PPARγ mRNA expression levels were assessed by relative quantification using qRT-PCR with SYBR Green I detection. Detection was done in a LightCycler® 96 System (Roche, Mannheim, Germany) and hypoxanthine-guanine phosphorribosyltransferase (HPRT) was used as endogenous control to normalize the results. A 10 min pre-incubation at 95°C was used followed by 45 cycles, of 3 step amplification, for 10 seconds at 95°C, 10 seconds at 59°C and 10 seconds at 72°C. The quantification reactions were carried out in a 96-well plate (Roche, Mannheim, Germany) containing 0.5µM of forward and reverse primers, 1X FastStart Essential DNA Green Master, and 3 µL of cDNA (47ng/µL). A duplicate for each cDNA sample was used as template for qRT-PCR.

The following primers for mice TH, PNMT, PPARƔ and HPRT were used: 5'-ACG TAC CCA TGT TGG CTG AC-3' (forward primer mice TH), 5'-CCA GTA CAC CGT GGA GAG TT-3' (reverse primer mice TH), 5'-CGCCTATCTCCGCAACAACT3' (forward primer mice PNMT), 5'- ACACCTCACCGGTAGCAAAG-3' (reverse primer mice PNMT), 5'-TGG GGA TGT CTC ACA ATG CAA-3' (forward primer mice PPARƔ), 5'-TTC CTG TCA CGA TCG CCC TCG-3' (reverse primer mice PPARƔ); 5' - AGC AGT ACA GCC CCA AAA TG-3' (forward primer mice HPRT), 5'- ATC CAA CAA AGT CTG GCC TGT-3' (reverse primer mice HPRT). Results were normalized with HPRT for each sample (-ΔCt).

### BrdU proliferation assay

3T3-L1 cells (1 × 10^5^ cells/mL) were cultured in 96-well plates with standard treatments in serum-free conditions for 24 h. Then, cells were labelled with BrdU at the final concentration of 10 μM for 12 h at 37°C. BrdU incorporation was quantified using the colorimetric Cell Proliferation ELISA, BrdU (Roche Diagnostics), according to the manufacturer's instructions. The results were normalized by dividing over the absorbance of the control treatment.

### Cell viability assay

3T3-L1 and both LPS-activated and non-activated RAW 264.7 macrophages cellular viability, after chronic treatment with the different CM, was assessed by MTT [3-(4,5-dimethylthiazol-2-yl)-2,5-diphenyl tetrazolium bromide] method (Sigma Aldrich, Germany) as previously described by Mosmann 18. The results were normalized as a percentage of the control treatment.

### *In vitro* treatments

3T3-L1 preadipocytes were seeded at a density of 4 000 cells/cm^2^ and kept in normal growth medium. Four days after adipogenic differentiation was induced and at day 10 of differentiation, cells were placed in the same medium lacking adipogenic MIX and dexamethasone and after placed, for 24 h, with CM from previously LPS-activated RAW 264.7 macrophages and RAW 264.7 macrophages. To investigate the chronic effect, preadipocytes, after reaching confluence, were also incubated with the same conditions during 24 h. Both LPS-activated RAW 264.7 and RAW 264.7 macrophages were exposed to CM from preadipocytes and mature adipocytes during 24 h. Preadipocytes and mature adipocytes were exposed, for 24 h, to three different conditions to compare with negative control medium (without stimuli): LPS at 500 ng/mL interleukin (IL) 4 at 10 ng/mL and interferon (IFN) γ at 10 ng/mL. This indirect coculture has the advantage of showing the effect of secreted factors without cellular interference.

### Statistical analysis

Results are expressed as mean ± standard error of the mean (SEM) or are summarized as median, 25^th^ and 75^th^ percentiles in a boxplot. Statistical significance of more than two groups was analyzed by one‐way analysis of variance (ANOVA), followed by Bonferroni's post-hoc test. A two-way ANOVA, followed by pairwise comparisons with Bonferroni's post-hoc test, was used to compare differences between preadipocytes and adipocytes, and differences between the several treatments (RAW 264.7 macrophages or LPS-activated RAW 264.7 macrophages, preadipocytes or mature adipocytes or cytokines). Normality of data distribution was assessed using the Shapiro-Wilk test, and the Levene's test for the homogeneity of variance. Statistical analyses were performed with the version 25.0 software Predictive Analytics Software (PASW Statistics Software) and Prism 6.0 software (Graph‐Pad Software Inc., La Jolla, CA) was used to plot the data. Significance was established at *p* < 0.05.

## Results

### TH and PMNT expression decreased along adipocyte differentiation

**Figure 1:**
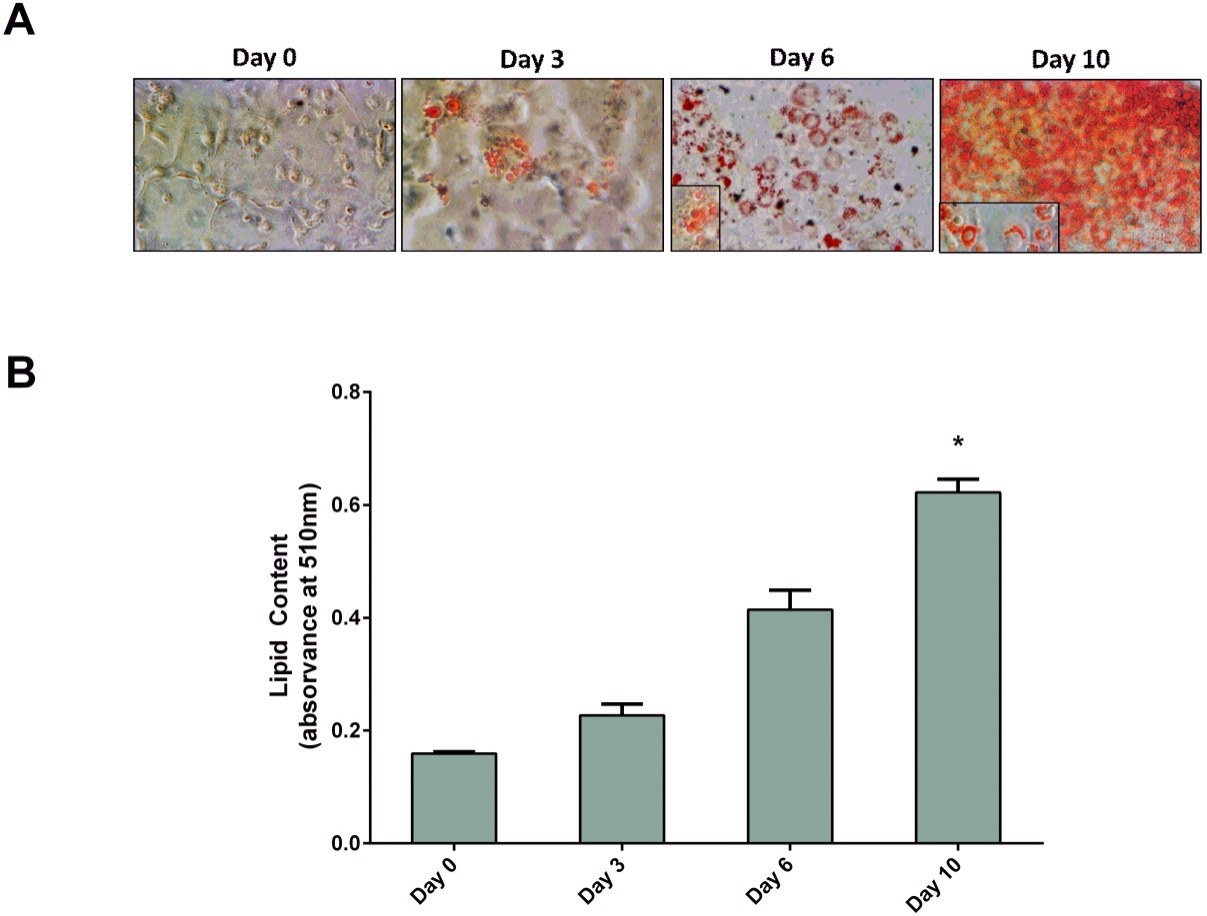
3T3-L1 adipocytes differentiation during 10 days. Cells were treated with supplemented culture medium to induce differentiation. Cells were stained with Oil-Red-O and photographed (A) and it was quantified the amount of Oil red O (n=6) (B). Results are presented as arithmetic mean value and SEM; *p < 0.05 vs day 0. Day 0 - day 0 of differentiation; Day 3 - day 3 of differentiation; Day 6 - day 6 of differentiation; Day 10 - day 10 of differentiation.

**Figure 2:**
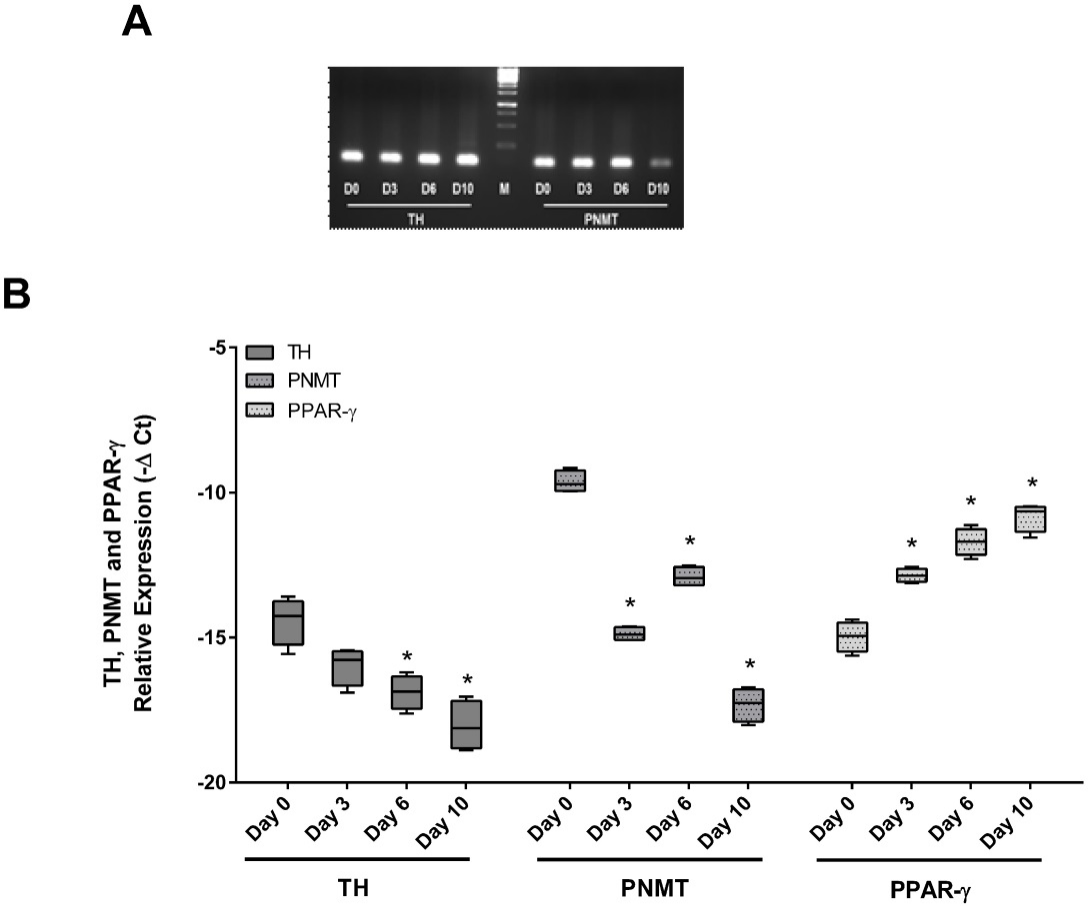
PCR amplification products for TH, PNMT and PPARγ. Identification of TH and PNMT by PCR amplification products with 110bp and 100bp bands, respectively (A). Normalized relative expression of TH, PNMT and PPARγ mRNA levels 3T3-L1 cell line (n=6) on different differentiation days (day 0, 3, 6 and 10) (B). *p < 0.05 vs respective day 0. Day 0 - day 0 of differentiation; Day 3 - day 3 of differentiation; Day 6 - day 6 of differentiation; Day 10 - day10 of differentiation. TH: tyrosine hydroxylase; PNMT: phenylethanolamine-N-methyl transferase; PPARγ: Peroxisome proliferator-activated receptor gamma; M: marker.

3T3-L1 cells were differentiated in culture into mature adipocytes. As seen in Figure 1, preadipocytes were fully differentiated at day 10. At this point, cells exhibited adipocyte-characteristic morphology and had a significant increase of lipid content, confirmed by the Oil Red O test, when compared with day 0 (Figure 1B). The expression of TH, PNMT and PPARγ along adipocyte differentiation was evaluated by qRT-PCR (Figure 2). Contrary to the PPARγ expression, both TH and PNMT expression decreased along the differentiation process (Figure 2B).

### CM from macrophages increases preadipocyte proliferation

**Figure 3:**
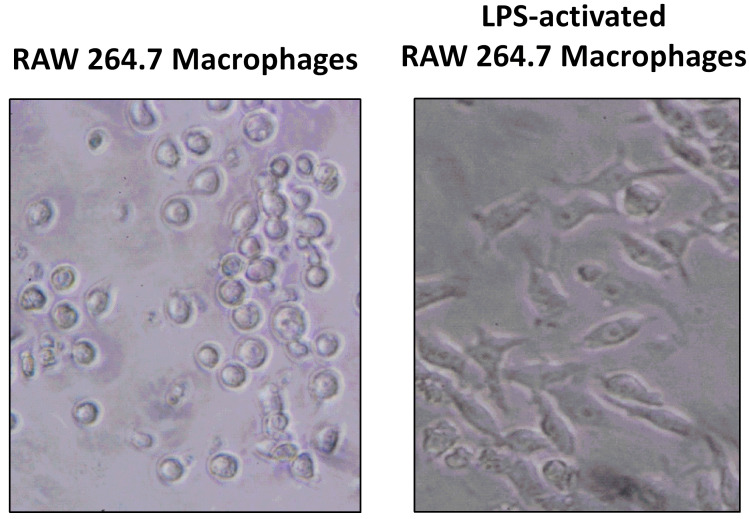
Raw 264.7 activation by lipopolysaccharide (LPS). Cells were incubated with media serum free (A) or with LPS (500 ng/mL) (B) during 24 h and then photographed (x100).

As explained before, we established a cell culture model where 3T3-L1 adipocytes were exposed to secretome from RAW 264.7 macrophages in order to study the effect of released inflammatory factors on the expression of TH and PNMT. Figure 3 shows that CM from LPS-activated macrophages was collected when more than 80% of activated macrophages was observed.

**Figure 4:**
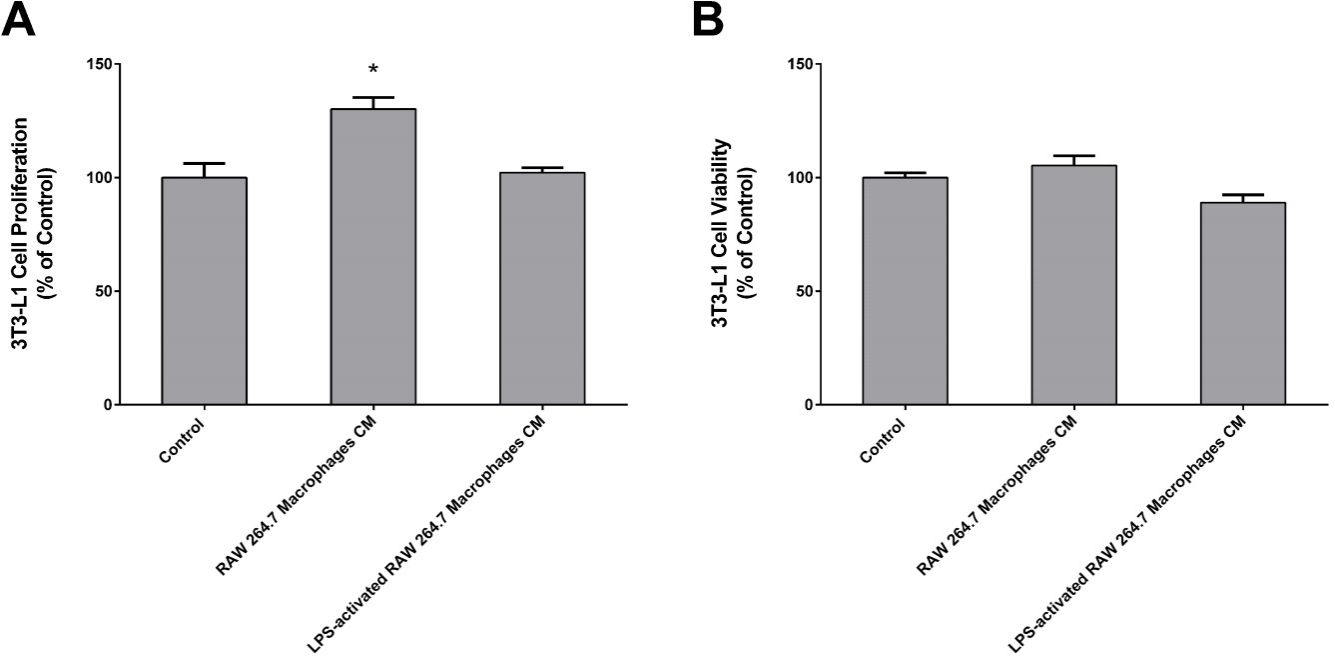
Chronic effect of Raw 264.7 secretome in 3T3-L1 proliferation (A) and viability (B). Both 3T3-L1 preadipocytes were incubated for 24 h with CM from both Raw 264.7 macrophages, LPS-activated RAW 264.7 macrophages and untreated (Control) (n=9) during 24 h. Results represent the percentage of viability or proliferating cells normalized over the absorbance of control (*p < 0.05 vs. Control).

3T3-L1 cells viability and proliferation was evaluated by the MTT and BrdU incorporation assays, respectively. There were no significant changes in 3T3-L1 cells viability after treatment with RAW CM (Figure 4B). However, 3T3-L1 cells proliferation was significantly increased in the presence of macrophages released factors (Figure 4A).

### CM from activated macrophages decreased the expression of TH and PNMT in adipocytes

**Figure 5:**
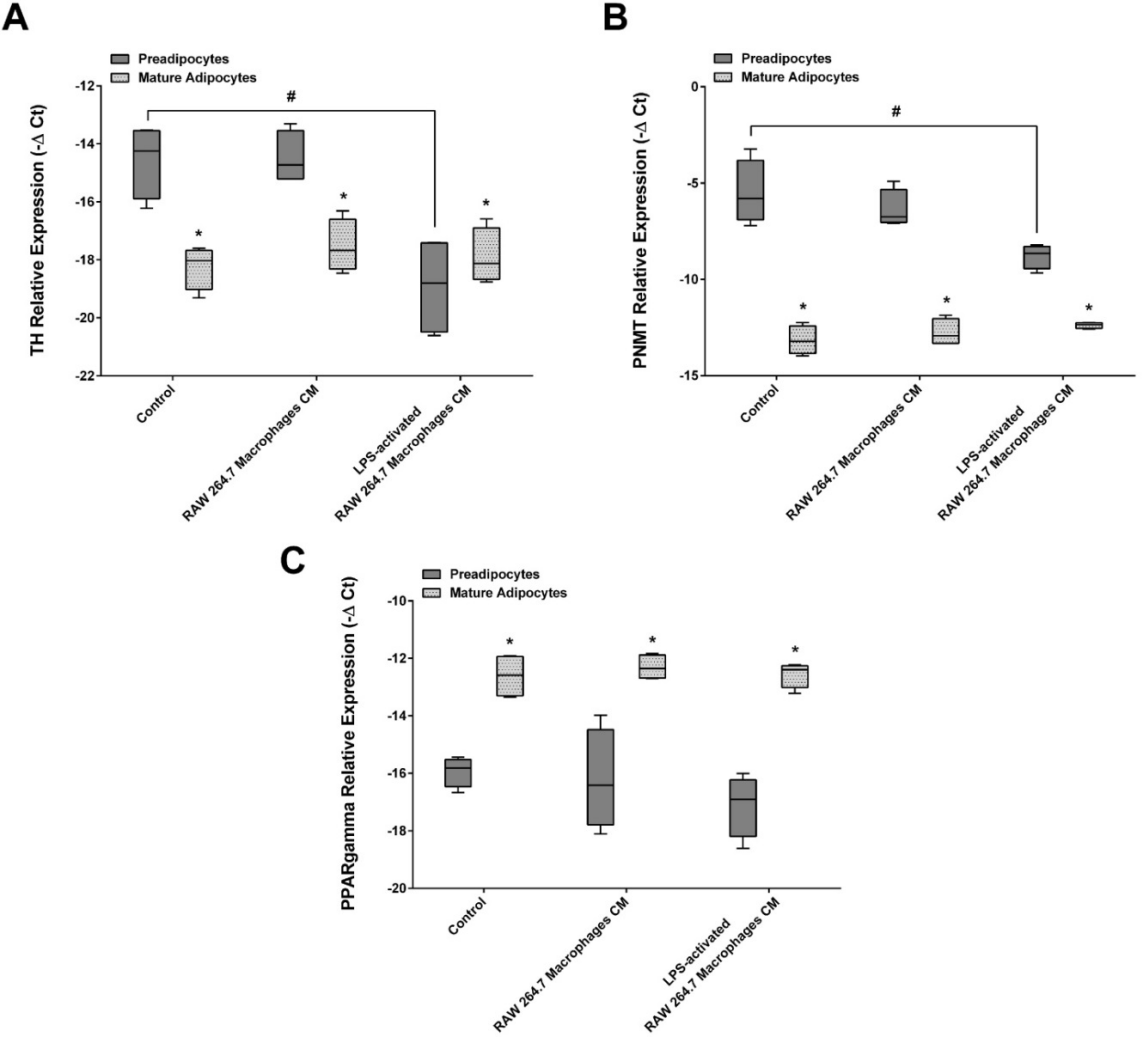
Effect of Raw 264.7 secretome in TH (A), PNMT (B) and PPARγ (C) gene expression by 3T3-L1 cells. 3T3-L1 preadipocytes and mature adipocytes were incubated for 24 h with CM from both Raw 264.7 macrophages, LPS-activated RAW 264.7 macrophages and untreated (Control) (n=9). *p < 0.05 preadipocytes vs mature adipocytes; #*p* < 0.05 preadipocytes treated vs preadipocytes control. TH: tyrosine hydroxylase; PNMT: phenylethanolamine-N-methyltransferase; PPARγ: peroxisome proliferator-activated receptor gamma.

We evaluated the chronic effect of CM from both non-activated and LPS-activated RAW 264.7 macrophages on TH, PNMT and PPARγ expression in preadipocytes and adipocytes. 3T3-L1 cell viability was not affected by CM but factors released by RAW 264.7 macrophages increased the proliferation of these cells to about 30% (Figure 4). Factors secreted by LPS-activated macrophages were able to decrease both TH and PNMT expression in preadipocytes. Interestingly, macrophage secretome did not significantly alter the expression of TH, PNMT or PPARγ in mature adipocytes (Figure 5A, 5B and 5C). However, mature adipocytes expressed less TH and PNMT than preadipocytes, while the expression of PPARγ increased along differentiation (Figure 5A, 5B and 5C).

### Adipocytes and preadipocytes: similar pattern of TH, PNMT and PPARγ expression after stimulation with pro and anti-inflammatory cytokines

**Figure 6:**
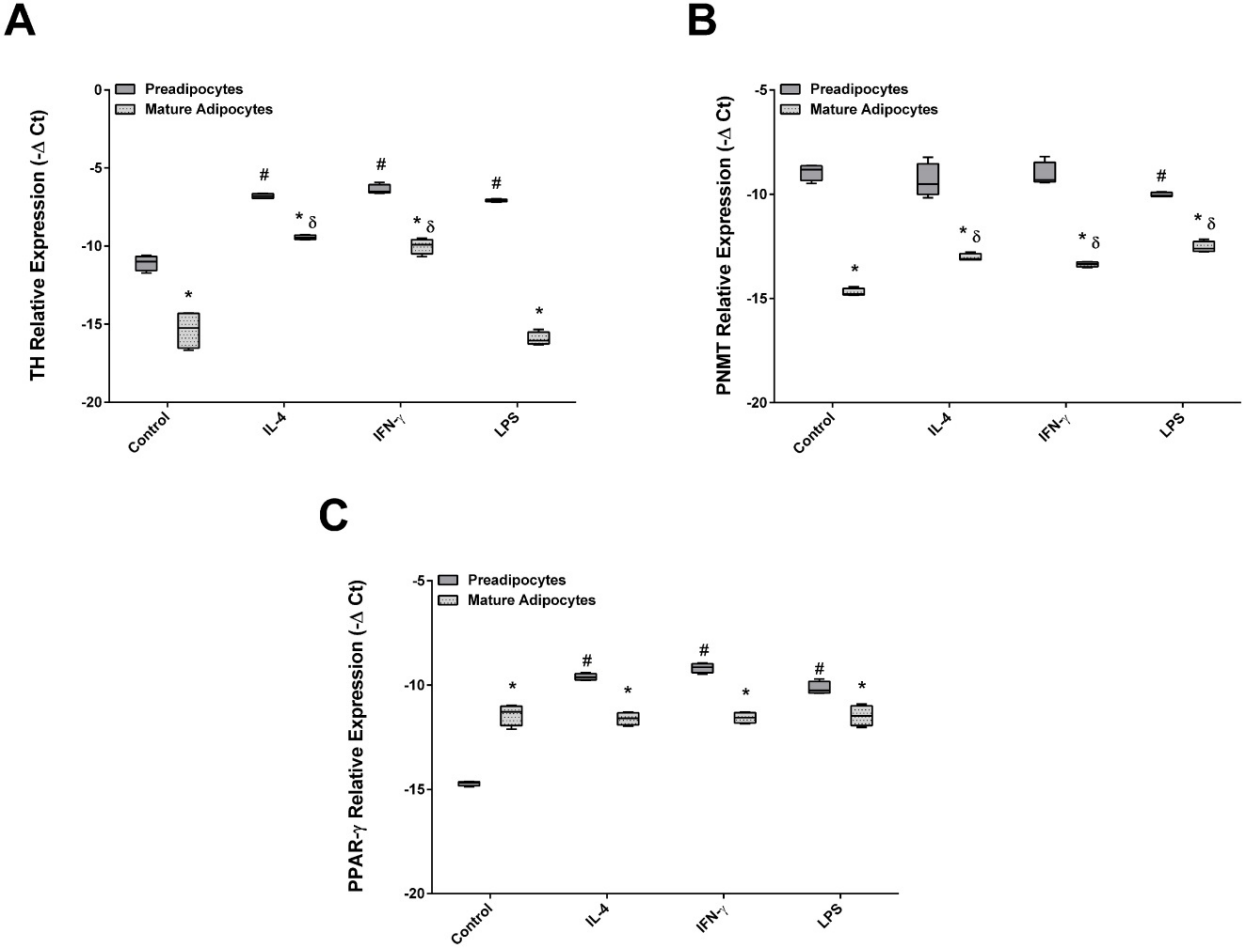
Effect of IL-4, IFN-γ and LPS in TH (A), PNMT (B) and PPARγ (C) gene expression by 3T3-L1 cells. 3T3-L1preadipocytes and adipocytes were incubated for 24 h with the different citokines and untreated (Control) (n=9). *p < 0.05 preadipocytes vs adipocytes; #*p* < 0.05 preadipocytes treated vs preadipocytes control, γ *p* < 0.05 adipocytes treated vs adipocytes control. TH: tyrosine hydroxylase; PNMT: phenylethanolamine-N-methyl transferase; PPARγ: peroxisome proliferator-activated receptor gamma.

Next, the effect of IFN-γ, IL-4 and the endotoxin LPS on TH, PNMT and PPARγ expression in both preadipocytes and mature adipocytes was studied (Figure 6). The pattern of TH, PNMT and PPARγ expression was found to be similar regardless of the agent used: mature adipocytes expressed lower levels of TH and PNMT and higher expression of PPARγ when compared with preadipocytes. Remarkably, treatment with pro and anti-inflammatory cytokines did not modify the pattern of TH, PNMT and PPARγ expression in both cells. Nevertheless, an increase of TH transcripts in preadipocytes was verified after stimulation with IL-4, IFN-γ or LPS comparing with control. Mature adipocytes exposed to IL-4 and IFN-γ, but not to LPS, showed an increase of TH expression. On the other hand, only LPS decreased PNMT mRNA in preadipocytes. Both studied cytokines had the same effect on PNMT and TH expression in mature adipocytes. The stimulation with both cytokines and LPS induced higher levels of PPARγ transcripts in preadipocytes but these remained unchanged in mature adipocytes (Figure 6A, B and C).

### Mature adipocytes CM decrease the expression of PNMT in activated macrophages

To further explore and deepen the study of the bidirectional cross-talk between adipocytes and macrophages, we reversed our experimental model. CM from 3T3-L1 preadipocytes and fully differentiated adipocytes was used to explore the potential effects of their secretome over TH and PNMT expression by both LPS-activated and non-activated macrophages.

**Figure 7:**
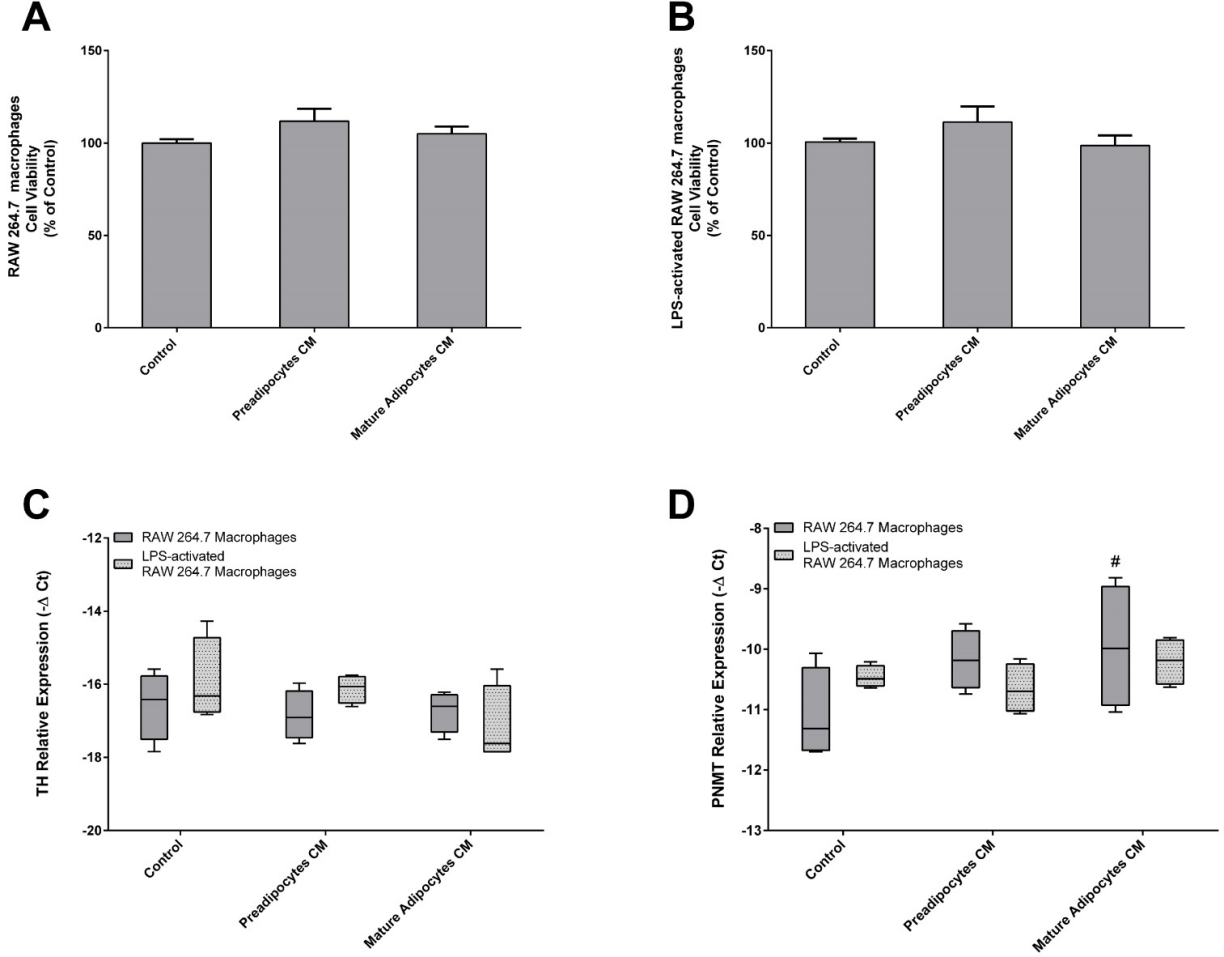
Effect of 3T3-L1 preadipocytes and mature adipocytes secretome in both Raw 264.7 macrophages (A) and LPS-activated RAW 264.7 macrophages (B) viability and in TH (C) and PNMT (D) gene expression by non- and activated Raw 264.7 cells. Raw 264.7 cells were incubated for 24 h with CM from both 3T3-L1 preadipocytes and adipocytes and untreated (Control) (n=6 and 9) after a treatment of 24 h. Results represent the percentage of viability or proliferating cells normalized over the absorbance of control (#*p* < 0.05 RAW 264.7 macrophages vs activated RAW 264.7 macrophages). TH: tyrosine hydroxylase; PNMT: phenylethanolamine-N-methyl transferase.

Initially, the viability of LPS-activated and non-activated RAW 264.7 macrophages was evaluated by the MTT assay (Figure 7A and B). Afterwards, we assessed the chronic effect of secreted factors from preadipocytes and mature adipocytes on the expression of TH and PMNT of both non- and LPS-activated macrophages (Figure 7C, D). The expression of TH in RAW 264.7 macrophages was affected neither by preadipocytes nor by mature adipocytes CM. However, factors secreted by mature adipocytes induced an increase of PNMT levels in RAW 264.7 macrophages when compared with activated macrophages. CM from pre and mature adipocytes did not influence the transcript levels of TH and PNMT in LPS-activated macrophages.

## Discussion

Obesity is characterized by a chronic subclinical inflammation that is thought to originate from AT and is linked to the progression of obesity-related comorbidities 19.

The close proximity of adipocytes and macrophages in obese AT *in vivo*, as the discovery that adipocytes also produce CA 20, triggered the interest for the bidirectional interaction between these cells. We very recently confirmed this result by describing for the first time the expression of TH and PNMT and CA production along the differentiation of adipocytes 21. The present study was undertaken to explore the expression of TH and PNMT in the crosstalk between adipocytes and macrophages, using an indirect coculture.

**Figure 8:**
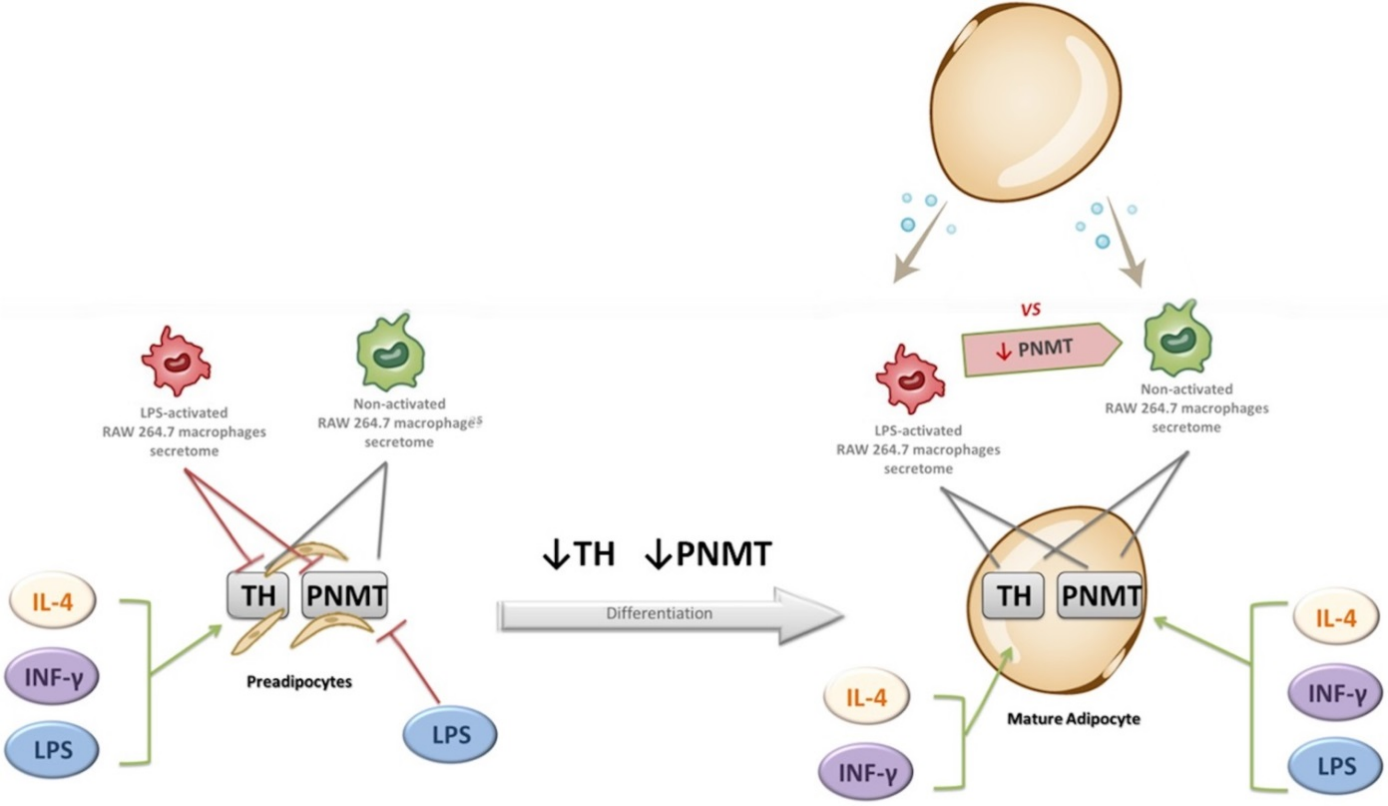
Effect of non-activated and LPS-activated RAW 264.7 macrophages secretome and of IL-4, IFN-γ and LPS upon the expression of TH and PNMT in preadipocytes and mature adipocytes. Green arrow: gene expression increase; Red arrow: gene expression decrease; Grey arrow: no effect. TH: tyrosine hydroxylase; PNMT: phenylethanolamine-N-methyl transferase.

The main findings of the study are fivefold: (1) the existence of a crosstalk between macrophages and adipocytes in the regulation of CA synthesis pathway; (2) a decreased expression of TH and PNMT during the adipocyte differentiation process; (3) a downregulation of TH and PNMT expression by CM from LPS-activated macrophages in preadipocytes, but not in mature adipocytes (4) a similar pattern of TH, PNMT and PPARγ expression after exposure to pro and anti-inflammatory cytokines in pre and mature adipocytes and (5) a decreased expression of PNMT in macrophages by CM from mature adipocytes, but not from preadipocytes. Figure 8 shows the main effects of macrophages secretome and pro and anti-inflammatory mediators on TH and PNMT gene expression in preadipocytes and mature adipocytes.

The present work showed that TH and PNMT expression decreases during adipocyte differentiation, as also during preadipocytes exposure to LPS-activated macrophages CM, while it was not affected in mature adipocytes. We may speculate that CA synthesis and downstream cellular fat regulation is more determinant in early stages of adipogenic differentiation due to the impact that inflammation might have on CA synthesis and their role in adipogenesis. For instance, NA stimulation of adipocytes induces downregulation of PPARγ among other white adipocytes marker genes 22. The expression of PPARγ in preadipocytes was diminished after both cytokines and LPS treatments, but was unaffected in mature adipocytes.

It is well known that LPS activated macrophages release higher levels of pro-inflammatory cytokines and less of anti-inflammatory adipokines/cytokines. In our study, LPS-activated macrophages secretome decreased the expression of enzymes related to CA synthesis in adipocytes. Some studies have been reporting the effect of pro-inflammatory mediators originated from macrophages secretome upon AT biology. It has been shown that inflammation triggers AT hypoxia in a model of 3T3-L1 adipocytes stimulated with CM from LPS- activated RAW 264.7 macrophages 23. LPS-stimulated RAW264.7 macrophages-derived IL-1β suppresses thermosgenesis due to its inhibition of β-AR-stimulated UCP1 induction in adipocytes 24. Although controversial 25, polarization of M2-like macrophages was reported to regulate thermogenesis and the browning of white AT via CA synthesis 26.

The upregulation of TH in adipocytes and preadipocytes by IFNγ and IL-4 stimulation may reflect an increase of CA synthesis to counteract the inflammatory environment. Notably, IL-4 was also reported to increase TH expression in macrophages 10.

There was no observable difference in the expression pattern of TH, PNMT and PPARγ in preadipocytes and adipocytes after pro (INF-γ) or anti-inflammatory (IL-4) cytokines exposure. This is somehow intriguing since the two cytokines are placed at cardinal positions in the regulation of immune reactions 27. Although we cannot exclude the role of other pro and anti-inflammatory mediators, these results infer that non-inflammatory signaling pathways may be involved in the regulation of adipocyte CA synthesis and adipogenesis. A previous study showed IL-4 prime tissue macrophages to accumulate lipids from dying fat cells and upregulate the expression of Alox15, a molecule that produces endogenous PPARγ ligands and adipogenesis 28. In other study, IL-4 led to the inhibition of adipogenesis and lipid accumulation by 3T3-L1 and to the release of pro-inflammatory non-esterified fatty acids by mature adipocytes 29.

Several lines of evidence support a role for IFN-γ in mediating obesity metabolic complications, with some being affected by CA. IFN-γ promotes lipolysis of cultured mouse adipocytes 30, inhibits adipocytes differentiation 31 and also seems to modulate CA handling by decreasing TH activity in chromaffin cells 32 and TH expression and endogenous CA production during the activation of peripheral blood mononuclear cells 33.

The evidence below, showing a relationship between CA production and receptors in immune cells and more favorable metabolic and inflammatory patterns, reinforces a role for CA in the immune and metabolic crosstalk within adipose tissue.

Besides the regulation of lipolysis 34, NA and AD also affect the remodeling of adipocytes 35, 36 and adipokines secretion 37. Β2Ars are highly expressed on immune cells and their activation by CA usually results in anti-inflammatory responses 38. Β2AR expression is associated with a better metabolic profile, with lower levels of leptin and with a lower inflammatory phenotype of monocytes 39. In addition, the expression of β2AR was shown to be down-regulated by TLR4 ligands, LPS and fatty acids stimulation 40 and downstream activation of the nuclear factor-kappa B (NF-kB) and therefore lower expression of pro-inflammatory cytokines.

The presence of dopaminergic receptors in both immune cells 41 and adipocytes 11 propose a regulatory role also for DA in this cellular crosstalk. Indeed, in line with other studies showing a role of CA in counteracting inflammation 12, 42, our group recently reported DA regulate human monocytes reduction of IL-6-induced signal transducer and activator of transcription 3 (STAT3) phosphorylation 43. DA seems to interfere with the production of TNF-α and nitric oxide in mouse monocytes and with the expression of surface markers involved in host defense 12 as also inhibits leptin release from human adipocytes 11. Visceral AT adipocytes, regarded as more inflammatory, express 10-fold greater DR D2 protein than subcutaneous AT 44 and in mouse 3T3-L1 adipocytes stimulation of DR D2 upregulated the expression of leptin and IL-6 44. In opposition, peripheral blood mononuclear cells expression of several subtypes of DR (and to a lesser extent also of TH) associated with lower weight, with better metabolic profile and with lower inflammation 7 and DR D2 expression in these cells seem to protect against central obesity 7.

CM from mature adipocytes, but not from preadipocytes, decreased the expression of PNMT in macrophages. Such result probably reflects the described differences in the secretome of adipocytes vs. preadipocytes 45 since adipokines are mainly produced by mature adipocytes. This also establishes a crosstalk between adipocytes and macrophages in the regulation of adrenaline synthesis. Altogether, these results suggest that the mediators released by macrophages seem to equally affect CA production by adipocytes, while adipocytes secretome preferentially affect adrenaline production in macrophages.

AT macrophages can be broadly classified as exhibiting either an M1 or M2 phenotype. The M1 phenotype induced by proinflammatory mediators such as INF-γ, TNFα and LPS is characterized by high production of pro-inflammatory cytokines, whereas the M2 phenotype mediates anti-inflammatory responses 46. Pertinent to our results is the demonstration that CA promotes M2 macrophage phenotype through β2 receptors 47 since the reduction of adrenaline production by macrophages here found might counteract its protective role in inflammatory obesity. Notably, in a previous work we have shown that adrenaline associated with a less inflammatory phenotype of monocytes 43.

LPS, known to possess highly potent pro-inflammatory effects, was also used to provoke a direct inflammatory response in adipocytes. In spite of the molecular mechanisms underlying LPS-regulated obesity remain largely unknown, some works have been describing its effect upon the expression of genes involved in inflammatory obesity 48-51. Interestingly enough, in our study LPS induced an opposite effect upon PNMT gene expression in preadipocytes, a finding that requires further investigation as once more after an inflammatory stimulus the synthesis of adrenaline appear to become impaired. As previously highlighted, the expression of PNMT was significantly reduced in macrophages and preadipocytes after exposure to adipocytes secretome and LPS, respectively. These observations support the notion that in obesity peripheral adrenaline is a preferred target for inflammatory mediators.

Our study has some limitations. Protein expression and activity are to be confirmed in future studies as we only measured gene expression.

## Conclusions

A model of indirect coculture of macrophages and adipocytes showed coregulation of the expression of CA synthesis enzymes through secretome. Might targeting chronic adipose inflammation by modulating CA synthesis or degradation machinery and/or blocking their receptors improve systemic metabolism in obesity? Our findings offer novel opportunities for revealing new AT therapeutic targets that could reverse its dysfunction in obesity. Dopaminergic and adrenergic agents could represent attractive candidates for this immunomodulation in inflammatory obesity.

## References

[1] Milagro FI, Mansego ML, De Miguel C, Martínez JA (2013). Dietary factors, epigenetic modifications and obesity outcomes: Progresses and perspectives. Molecular Aspects of Medicine.

[2] van der Klaauw Agatha A, Farooqi IS (2015). The Hunger Genes: Pathways to Obesity. Cell.

[3] Medzhitov R (2008). Origin and physiological roles of inflammation. Nature.

[4] Skurk T, Alberti-Huber C, Herder C, Hauner H (2007). Relationship between Adipocyte Size and Adipokine Expression and Secretion. The Journal of Clinical Endocrinology & Metabolism.

[5] Jernås M, Palming J, Sjöholm K, Jennische E, Svensson P-A, Gabrielsson BG (2006). Separation of human adipocytes by size: hypertrophic fat cells display distinct gene expression. The FASEB Journal.

[6] Hotamisligil GS (2017). Inflammation, metaflammation and immunometabolic disorders. Nature.

[7] Leite F, Lima M, Marino F, Cosentino M, Ribeiro L (2016). Dopaminergic Receptors and Tyrosine Hydroxylase Expression in Peripheral Blood Mononuclear Cells: A Distinct Pattern in Central Obesity. PloS one.

[8] Ouchi N, Parker JL, Lugus JJ, Walsh K (2011). Adipokines in inflammation and metabolic disease. Nature Reviews Immunology.

[9] Than A, Ye F, Xue R, Ong JW, Poh CL, Chen P (2011). The crosstalks between adipokines and catecholamines. Molecular and Cellular Endocrinology.

[10] Vargovic P, Laukova M, Ukropec J, Manz G, Kvetnansky R (2016). Lipopolysaccharide induces catecholamine production in mesenteric adipose tissue of rats previously exposed to immobilization stress. Stress.

[11] Borcherding DC, Hugo ER, Idelman G, De Silva A, Richtand NW, Loftus J (2011). Dopamine receptors in human adipocytes: expression and functions. PloS one.

[12] Pinoli M, Marino F, Cosentino M (2017). Dopaminergic Regulation of Innate Immunity: a Review. Journal of Neuroimmune Pharmacology.

[13] Arreola R, Alvarez-Herrera S, Pérez-Sánchez G, Becerril-Villanueva E, Cruz-Fuentes C, Flores-Gutierrez EO (2016). Immunomodulatory Effects Mediated by Dopamine. Journal of Immunology Research.

[14] Chou R, Stinson M, Noble B, Spengler R (1996). Beta-adrenergic receptor regulation of macrophage-derived tumor necrosis factor-alpha production from rats with experimental arthritis. Journal of Neuroimmunology.

[15] Flierl MA, Rittirsch D, Huber-Lang M, Sarma JV, Ward PA (2008). Catecholamines - Crafty Weapons in the Inflammatory Arsenal of Immune/Inflammatory Cells or Opening Pandora's Box?. Molecular Medicine.

[16] Mosser DM, Zhang X (2008). Activation of Murine Macrophages. Current protocols in immunology/edited by John E Coligan [et al].

[17] Lumeng CN, Bodzin JL, Saltiel AR (2007). Obesity induces a phenotypic switch in adipose tissue macrophage polarization. Journal of Clinical Investigation.

[18] Mosmann T (1983). Rapid colorimetric assay for cellular growth and survival: Application to proliferation and cytotoxicity assays. Journal of Immunological Methods.

[19] Kershaw EE, Flier JS (2004). Adipose Tissue as an Endocrine Organ. The Journal of Clinical Endocrinology & Metabolism.

[20] Vargovic P, Ukropec J, Laukova M, Cleary S, Manz B, Pacak K (2011). Adipocytes as a new source of catecholamine production. FEBS Letters.

[21] Gomes A, Soares R, Costa R, Marino F, Cosentino M, Malagon MM (2020). High-fat diet promotes adrenaline production by visceral adipocytes. European Journal of Nutrition.

[22] Higareda-Almaraz JC, Karbiener M, Giroud M, Pauler FM, Gerhalter T, Herzig S (2018). Norepinephrine triggers an immediate-early regulatory network response in primary human white adipocytes. BMC genomics.

[23] Lopez-Pascual A, Lorente-Cebrián S, Moreno-Aliaga MJ, Martinez JA, González-Muniesa P (2019). Inflammation stimulates hypoxia-inducible factor-1α regulatory activity in 3T3-L1 adipocytes with conditioned medium from lipopolysaccharide-activated RAW 264.7 macrophages. Journal of Cellular Physiology.

[24] Goto T, Naknukool S, Yoshitake R, Hanafusa Y, Tokiwa S, Li Y (2016). Proinflammatory cytokine interleukin-1β suppresses cold-induced thermogenesis in adipocytes. Cytokine.

[25] Fischer K, Ruiz HH, Jhun K, Finan B, Oberlin DJ, van der Heide V (2017). Alternatively activated macrophages do not synthesize catecholamines or contribute to adipose tissue adaptive thermogenesis. Nature Medicine.

[26] Igarashi Y, Nawaz A, Kado T, Bilal M, Kuwano T, Yamamoto S (2018). Partial depletion of CD206-positive M2-like macrophages induces proliferation of beige progenitors and enhances browning after cold stimulation. Scientific Reports.

[27] Paludan (1998). Interleukin-4 and Interferon-γ: The Quintessence of a Mutual Antagonistic Relationship. Scandinavian Journal of Immunology.

[28] Lee Y-H, Kim S-N, Kwon H-J, Maddipati KR, Granneman JG (2016). Adipogenic role of alternatively activated macrophages in β-adrenergic remodeling of white adipose tissue. American Journal of Physiology Regulatory, Integrative and Comparative Physiology.

[29] Tsao C-H, Shiau M-Y, Chuang P-H, Chang Y-H, Hwang J (2014). Interleukin-4 regulates lipid metabolism by inhibiting adipogenesis and promoting lipolysis. Journal of Lipid Research.

[30] Benelli C, Distel E, Collinet M, Khazen W, Forest C, Chaves VrE (2007). Acute and Selective Inhibition of Adipocyte Glyceroneogenesis and Cytosolic Phosphoenolpyruvate Carboxykinase by Interferon γ. Endocrinology.

[31] McGillicuddy FC, Chiquoine EH, Hinkle CC, Kim RJ, Shah R, Roche HM (2009). Interferon gamma attenuates insulin signaling, lipid storage, and differentiation in human adipocytes via activation of the JAK/STAT pathway. The Journal of Biological Chemistry.

[32] Anastasiadis PZ, Kuhn DM, Blitz J, Imerman BA, Louie MC, Levine RA (1996). Regulation of tyrosine hydroxylase and tetrahydrobiopterin biosynthetic enzymes in PC12 cells by NGF, EGF and IFN-γ. Brain Research.

[33] Cosentino M, Zaffaroni M, Ferrari M, Marino F, Bombelli R, Rasini E (2005). Interferon-gamma and interferon-beta affect endogenous catecholamines in human peripheral blood mononuclear cells: Implications for multiple sclerosis. Journal of Neuroimmunology.

[34] Lafontan M, Langin D (2009). Lipolysis and lipid mobilization in human adipose tissue. Progress in Lipid Research.

[35] Zhu X, He Q, Lin Z (2003). [Effects of catecholamines on human preadipocyte proliferation and differentiation]. Zhonghua Zheng Xing Wai Ke Za Zhi.

[36] Bowers RR, Festuccia WTL, Song CK, Shi H, Migliorini RH, Bartness TJ (2004). Sympathetic innervation of white adipose tissue and its regulation of fat cell number. American Journal of Physiology-Regulatory, Integrative and Comparative Physiology.

[37] Flierl MA, Rittirsch D, Nadeau BA, Chen AJ, Sarma JV, Zetoune FS (2007). Phagocyte-derived catecholamines enhance acute inflammatory injury. Nature.

[38] Bosmann M, Grailer JJ, Zhu K, Matthay MA, Sarma JV, Zetoune FS (2012). Anti-inflammatory effects of β2 adrenergic receptor agonists in experimental acute lung injury. FASEB J.

[39] Leite F, Lima M, Marino F, Cosentino M, Ribeiro L (2017). β(2) Adrenoceptors are underexpressed in peripheral blood mononuclear cells and associated with a better metabolic profile in central obesity. International Journal of Medical Sciences.

[40] Kizaki T, Izawa T, Sakurai T, Haga S, Taniguchi N, Tajiri H (2008). Beta2-adrenergic receptor regulates Toll-like receptor-4-induced nuclear factor-kappaB activation through beta-arrestin 2. Immunology.

[41] Talhada D, Rabenstein M, Ruscher K (2018). The role of dopaminergic immune cell signalling in poststroke inflammation. Therapeutic advances in neurological disorders.

[42] Camell CD, Sander J, Spadaro O, Lee A, Nguyen KY, Wing A (2017). Inflammasome-driven catecholamine catabolism in macrophages blunts lipolysis during ageing. Nature.

[43] Leite F, Leite Â, Rasini E, Gaiazzi M, Ribeiro L, Marino F (2018). Dopaminergic pathways in obesity-associated immuno-metabolic depression. Psychological Medicine.

[44] Wang X, Villar VA, Tiu A, Upadhyay KK, Cuevas S (2018). Dopamine D2 receptor upregulates leptin and IL-6 in adipocytes. Journal of Lipid Research.

[45] Ojima K, Oe M, Nakajima I, Muroya S, Nishimura T (2016). Dynamics of protein secretion during adipocyte differentiation. FEBS Open Bio.

[46] Gordon S, Martinez FO (2010). Alternative Activation of Macrophages: Mechanism and Functions. Immunity.

[47] Grailer JJ, Haggadone MD, Sarma JV, Zetoune FS, Ward PA (2014). Induction of M2 regulatory macrophages through the β2-adrenergic receptor with protection during endotoxemia and acute lung injury. Journal of Innate Immunity.

[48] Chirumbolo S, Franceschetti G, Zoico E, Bambace C, Cominacini L, Zamboni M (2014). LPS response pattern of inflammatory adipokines in an *in vitro* 3T3-L1 murine adipocyte model. Inflammation Research.

[49] Unno Y, Sato Y, Nishida S, Nakano A, Nakano R, Ubagai T (2017). Acinetobacter baumannii Lipopolysaccharide Influences Adipokine Expression in 3T3-L1 Adipocytes. Mediators Inflammation.

[50] Lu B, Lu Y, Moser AH, Shigenaga JK, Grunfeld C, Feingold KR (2008). LPS and proinflammatory cytokines decrease lipin-1 in mouse adipose tissue and 3T3-L1 adipocytes. American Journal of Physiology-Endocrinology and Metabolism.

[51] Chang C-C, Sia K-C, Chang J-F, Lin C-M, Yang C-M, Huang K-Y (2019). Lipopolysaccharide promoted proliferation and adipogenesis of preadipocytes through JAK/STAT and AMPK-regulated cPLA2 expression. International Journal of Medical Sciences.

